# An enhanced approach to simulation-based mastery learning: optimising the educational impact of a novel, National Postgraduate Medical Boot Camp

**DOI:** 10.1186/s41077-021-00157-1

**Published:** 2021-04-26

**Authors:** Emma L. Scahill, Nathan G. Oliver, Victoria R. Tallentire, Simon Edgar, James F. Tiernan

**Affiliations:** Medical Education Directorate, NHS Lothian, Edinburgh, UK

**Keywords:** Simulation-based mastery learning (SBML), Pre-learning, Peer-assisted deliberate practice, Peer observation

## Abstract

**Background:**

Simulation-based mastery learning (SBML) is an effective, evidence-based methodology for procedural skill acquisition, but its application may be limited by its resource intensive nature. To address this issue, an enhanced SBML programme has been developed by the addition of both pre-learning and peer learning components. These components allowed the enhanced programme to be scaled up and delivered to 106 postgraduate doctors participating in a national educational teaching programme.

**Methods:**

The pre-learning component consisted of an online reading pack and videos. The peer learning component consisted of peer-assisted deliberate practice and peer observation of assessment and feedback within the SBML session. Anonymised pre- and post-course questionnaires were completed by learners who participated in the enhanced programme. A mixture of quantitative and qualitative data was obtained.

**Results:**

Questionnaires were distributed to and completed by 50 learners. Both sections of the pre-learning component were highly rated on the basis of a seven-point Likert scale. The peer learning component was also favourably received following a Likert scale rating. Peer observation of the performance and assessment process was rated similarly by first and second learners.

The thematic analysis of the reasons for which peer-assisted deliberate practice was considered useful showed that familiarisation with equipment, the rehearsal of the procedure itself, the exchange of experiences and sharing of useful tips were important. The thematic analysis of the reasons why peer observation during ‘performance, assessment and feedback’ was useful highlighted that an ability to compare a peer’s performance to their own and learning from observing a peer’s mistakes were particularly helpful.

**Conclusion:**

The SBML programme described has been enhanced by the addition of pre-learning and peer learning components which are educationally valued and allow its application on a national scale.

**Supplementary Information:**

The online version contains supplementary material available at 10.1186/s41077-021-00157-1.

## Introduction

### Background

Simulation-based mastery learning (SBML) is a robust, evidence-based teaching methodology designed to improve the acquisition of procedural skills, with the aim of supporting all learners to achieve an agreed standard [1]. SBML programmes have been established around a growing number of high-risk clinical procedures, and the literature has consistently demonstrated a favourable impact on complication rates and cost savings [2, 3]. There is clear evidence to suggest that SBML is superior to traditional methods of teaching procedural skills [4]. The established methodological approach to SBML, regardless of the clinical skill, typically comprises three phases. Firstly, a pre-testing phase which establishes the learners’ baseline knowledge and skill set. Secondly, an educational phase often including a tutor demonstration as well as a deliberate practice. Thirdly, a test phase during which the learner demonstrates ‘competency’ according to a validated checklist [5, 6]. This established methodological approach to SBML has not been reported to include a pre-learning component. The absence of such a component is likely to increase the requirement for faculty input throughout the process, and this may have resource implications which could prove prohibitive in a teaching programme of this type.

Whilst the evidence for the educational impact of SBML is persuasive, effective implementation of the methodology in the context of limited resources is challenging. To optimise provision of SBML opportunities for postgraduate doctors-in-training, a novel mastery learning programme was created. This adapted the established methodological approach to SBML by elimination of the pre-testing phase and enhanced the process through the incorporation of new components, including online pre-learning resources and peer learning. The enhanced programme was first devised and embedded into postgraduate medical education by the Medical Education Directorate of NHS Lothian [7]. It was subsequently adopted by the Scottish Postgraduate Deanery (NHS Education for Scotland) for Internal Medicine Training (IMT) and collaboratively expanded across all regions of the country.

The enhanced programme contains two components, pre-learning and peer learning (Additional file 1). The pre-learning component consists of reading and video sections. The peer learning consists of two sections. Firstly, a peer assisted deliberate practice section and secondly peer observation of the assessment and feedback delivered to a fellow learner either before or after performing the procedure themselves. This enhanced programme was implemented and evaluated in the context of a national postgraduate medical programme in Scotland in 2019. Questionnaires, for completion by the learners, were designed with the intention of identifying the components of the enhanced programme which were most useful and the reasons for this. Analysis of the data generated will continue to inform further development of the enhanced Mastery programme.

The initial impetus to expand this enhanced SBML programme across Scotland was pragmatic. In August 2019, a new Internal Medicine Training curriculum [8], comprising a range of procedural skill competencies, was introduced for the UK medical trainees. Scotland uniquely embraced this challenge by creating an ‘IMT boot camp’, utilising simulation methodology to address many curricular requirements. The IMT boot camp approach employs the principles of SBML and applies them to the procedural components which include lumbar puncture (LP) and ascitic and pleural procedures. The main challenge of this approach was the efficient provision of high-quality, reproducible training for 106 postgraduate trainees who commenced IMT in August 2019.

### Building an enhanced SBML programme

The group researched educational-based frameworks and created a new programme to mastery-based competency through an iterative series of meetings and workshops with local educationalists and subject matter experts. The ‘scaffolding’ and ‘social learning’ theories were identified as being most appropriate to the development of the enhanced programme [9, 10].

### Pre-learning

Pre-learning reading packs and videos were produced to provide the learners with background knowledge and an understanding of the procedure prior to attending the skills lab session. This approach was based on the ‘scaffolding’ theory of educational constructivist Bruner [9]. ‘Scaffolding’ refers to the process whereby educators engage learners in a collection of meaningful learning activities, arranged in a sequence that provides a platform, or scaffold, of increasingly complex pieces of knowledge or skills [11]. It was intended that the creation of a ‘scaffolded’ SBML programme would enable learners to reach the upper limits of their ability more quickly having been better prepared for their skills lab session. This is particularly relevant when learning to perform complex procedures such as lumbar puncture [11].

The use of educational videos has increased over the last decade [12]. E-learning, involving online learning based on computers, has been shown to have a positive effect on both learning delivery and enhancing the learning [13]. Henriksen et al. [14] reported that residents who watched a learner centred instructional video performed the procedure of LP to a higher standard than those given written instructions. Srivastava et al. [15] reported that learners felt more comfortable and competent when performing LPs on paediatric patients after watching an instructional video. These authors also reported uptake of best LP practices which were demonstrated in an exemplar video. Cheung et al. [16] investigated the role of preparatory materials in SBML for central venous catheter insertion by medical students. Their study demonstrated that learners who prepared using web-based videos of observational practice and reading packs completed the SBML workshop in less time than those who prepared using a reading pack alone.

The implementation of the self-paced pre-learning element of the mastery programme prior to the skills lab simulation sessions employs the ‘flipped classroom’ approach which is a blended learning method widely used in medical education [17].

### Peer learning

Peer-assisted deliberate practice, a form of peer-learning, encompasses the process whereby one student learns from another [18]. Bandura [10] described learning as a socially influenced phenomenon and stated that human behaviour was shaped through social interactions and observations. Bandura’s ‘social learning theory’ provided the theoretical basis for the addition of the peer-assisted practice and peer observation sections to the SBML programme. Horburgh and Ippolito described peer learning based on co-learners observing and working together in the context of clinical practice [19]. Same level peer learning has been shown to improve collaboration, knowledge and evaluation and procedural skills [20]. Peer-assisted deliberate practice and peer observation sections were incorporated into the SBML process for these reasons.

### Aims

1. To develop an enhanced mastery programme which improves the educational experience for the learner and increases the efficiency of delivery of a novel, national educational programme.
2. To identify the aspects of the enhanced programme that are considered most useful to learners and to explore the underlying reasons.

## Methods

### Pre-learning

The lumbar puncture procedure will be used as an example of the enhancement of the SBML programme.

A pre-reading LP pack was made available online for the boot camp participants several weeks in advance. Participants had the option to download the reading pack onto their personal or work devices for asynchronous learning. The pack consisted of a comprehensive, evidence-based document compiled by a core group of physicians and anaesthetists which had been quality assured through an iterative process conducted by a selection of subject matter experts. The reading pack included an overview of the mastery skills programme (Additional file 1), learning outcomes, indications and risk assessment, patient safety considerations and potential complications. It also included a brief overview of the relevant anatomy, physiology and equipment. The procedure was divided into six procedural phases (Additional file 2), and the SBML LP assessment checklist was included (Additional file 3).

Two videos, whose design was consistent with the principles described by Dong and Goh, were included in the pre-learning [12]. The first video was 20 min in duration and featured an exemplar skill performance of lumbar puncture. This video was filmed in the skills lab and features a simulated patient with a part-task simulation trainer for the procedure itself. The procedure was aligned to the six phases described in the reading pack. These phases, used as a framework for all the SBML resources, were ‘Preparation, Assistance and Positioning’, ‘Procedural Pause’, ‘Asepsis and Anaesthetic’, ‘Insertion’, ‘Anchoring and Dressing’ and finally ‘Completion’ (Additional file 2).

The second video, with a duration of 30 min, was an example of a mastery skills session. It demonstrated ‘what will happen on the day’ and shows the learner making some common errors during the procedure, receiving feedback and then repeating the procedure to a higher standard before passing the assessment. Completion of the pre-learning was highlighted as being mandatory for attendance at the boot camp. Each of the boot camp attendees reported that they had completed the LP pre-learning.

### Peer learning

#### Peer-assisted deliberate practice

A period of 2 h was allocated for the SBML LP session within the 3-day boot camp. Learners entered the lumbar puncture training suite in groups of six and were then placed in pairs before being randomly allocated to one of three members of faculty. The LP faculty included clinicians experienced in both lumbar puncture and the process of SBML. Each faculty member then demonstrated the equipment which had been made available to the learners. The pairs of learners were given time to familiarise themselves with the equipment and to practice the technical aspects of the procedure prior to their assessment. This constituted the peer-assisted deliberate practice section. During this period, the faculty had the option of observing their allocated pair of learners through one-way glass. They also had the opportunity for further informal discussion about the equipment or correction of the learners’ technique if they considered this desirable or necessary. The period of peer-assisted deliberate practice, which was an average of 20 min, was subsequently followed by a period of facilitator feedback and further practice if necessary. This ended when both learners in the pair felt that they were sufficiently familiar with the equipment and technique to be able to proceed to the next stage.

#### Peer observation of assessment and feedback

This commenced with the faculty member presenting the pair of learners with a basic clinical scenario which required them to perform a lumbar puncture on a patient. Each learner addressed the clinical scenario individually whilst the other member of the pair observed.

The faculty were provided with a checklist which detailed all of the points to be considered and actions to be taken by the learner being assessed. These included confirming the patient’s identity, reviewing the clinical findings, excluding contraindications and obtaining informed consent.

The faculty then acted as the learner’s ‘skilled assistant’ during the simulated lumbar puncture procedure. Following completion of a surgical scrub, the learners were required to include a safety pause before commencing the procedure and obtaining a sample of ‘cerebrospinal fluid’. Learners were assessed on their ability to perform the procedure safely and effectively whilst communicating with their assistant and the patient (represented by the LP part-task trainer). Learners were scored against a 23-point checklist (Additional file 3) created and standard set by a group of subject matter experts using a Mastery Angoff method [21].

Following each learner’s performance, the faculty member provided several minutes of feedback and determined whether or not the learner had completed the SBML LP to the required standard. If the standard was not met time was available for the learner to immediately repeat the performance, assessment and feedback process. Should the learner still not complete the procedure satisfactorily, arrangements were made for them to have further local training.

#### Data collection and analysis

A total of fifty learners were asked to complete both pre-course and post-course questionnaires. These related to the pre-learning and peer learning experiences respectively. The post-course questionnaires were completed immediately following the SBML LP session. All fifty questionnaires were returned; however, two of these were only partially completed but still taken into consideration. A combination of quantitative and qualitative data was collected.

Likert scales were used to generate quantitative data on all four sections of the enhanced programme. Qualitative data, in the form of short answers from the learners which explained their Likert scale rating, were obtained and subjected to a thematic analysis.

Thematic analysis was based on the approach of Braun and Clarke [22] and included the stages of familiarisation with data, generation of initial codes, searching for and reviewing themes, and defining themes. This process culminated in the learners’ responses being allocated to specific themes.

## Results

### Pre-learning

**Fig. 1:**
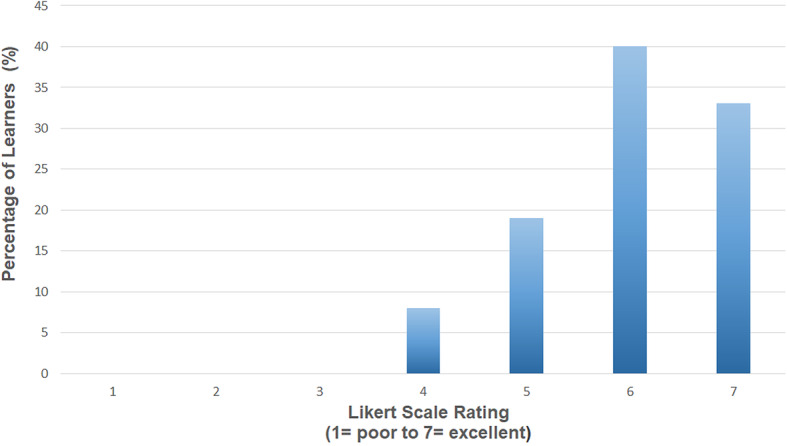
Learner rating of the pre-learning reading

**Fig. 2:**
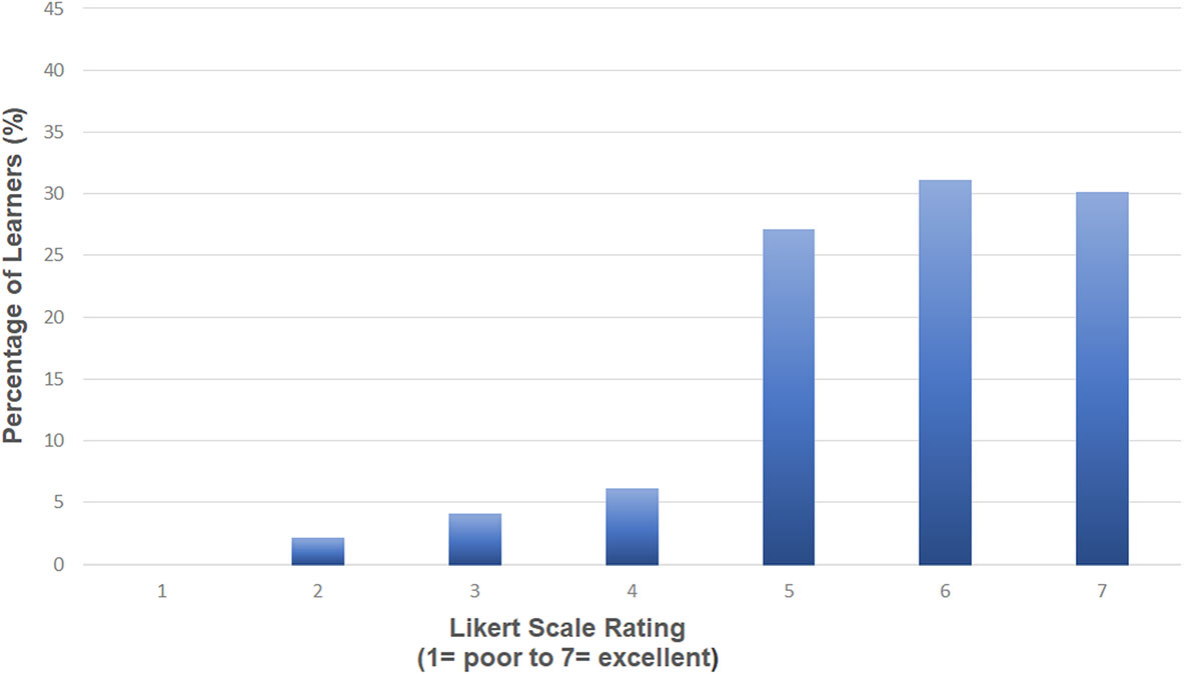
Learner rating of the pre-learning videos

The pre-learning reading pack and videos were rated by learners using a Likert scale (Figs. 1 and 2).

The results show that learners rated both aspects of the pre-learning highly.

### Peer Learning

#### Peer-assisted deliberate practice

**Fig. 3:**
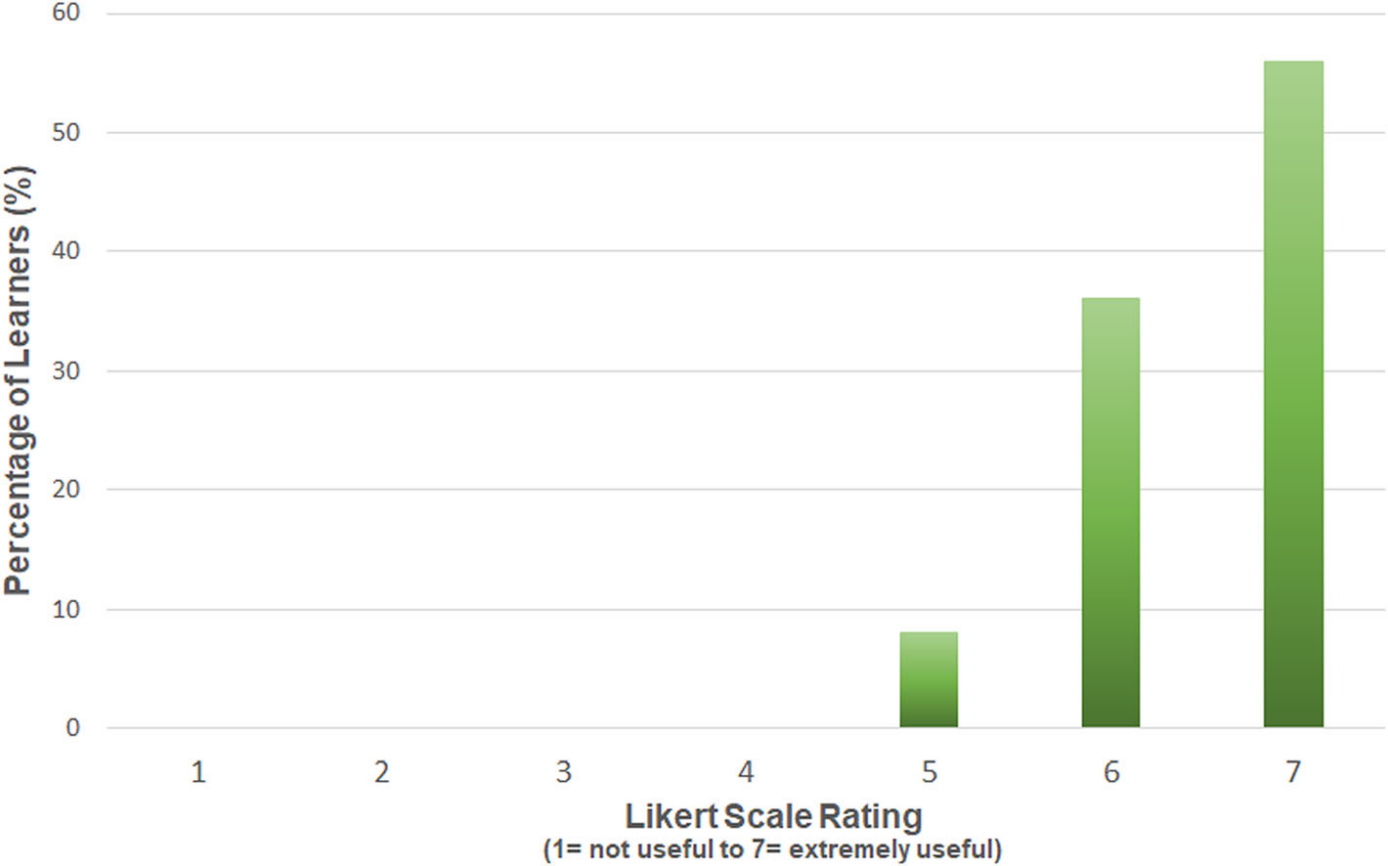
Learner-rated usefulness of peer-assisted deliberate practice

Learners rated the usefulness of peer-assisted deliberate practice using a Likert scale (Fig. 3). The results show that learners found the peer-assisted deliberate practice to be useful.

**Fig. 4:**
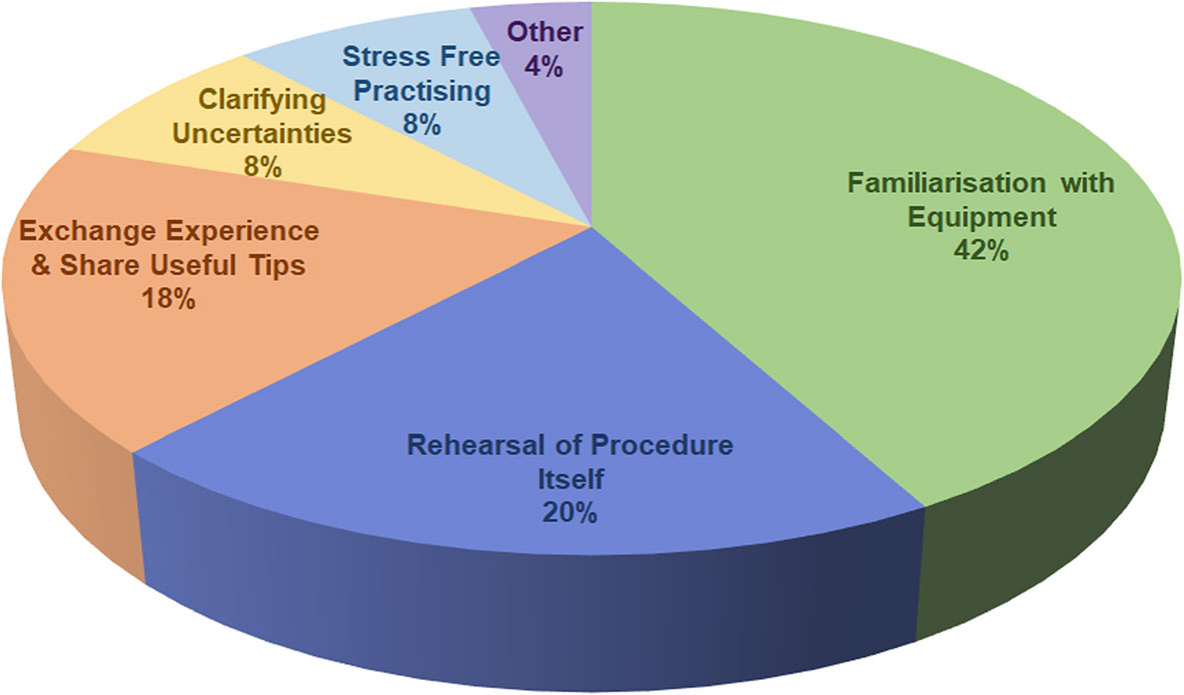
Themes indicating why the peer-assisted deliberate practice section was considered useful

Qualitative data demonstrating the reasons for the high usefulness rating were analysed and the resulting themes are presented in Fig. 4. Examples of learner quotes, according to the themes to which they were allocated, are shown in Table 1.

**Table 1:**
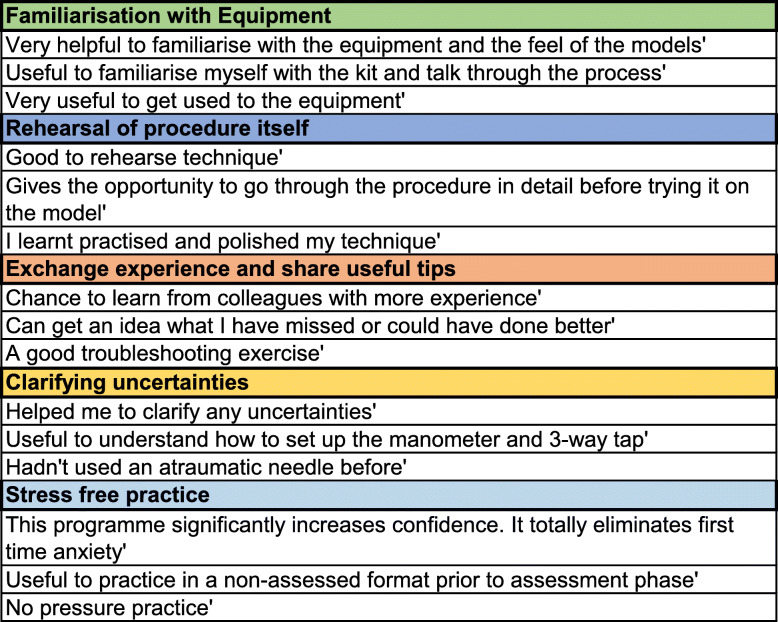
Peer-assisted deliberate practice: examples of learner quotes categorised by theme

#### Peer observation of assessment and feedback

**Fig. 5:**
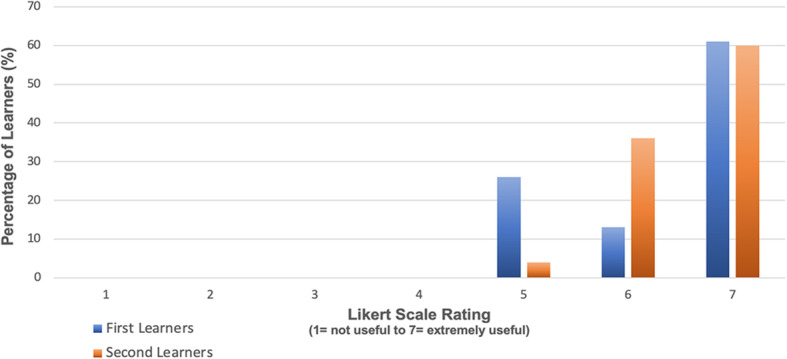
Comparison of 1st and 2nd learner usefulness ratings during peer observation of ‘assessment and feedback’

Likert scales were used to analyse the usefulness of learners observing their peer during the performance, assessment and feedback process (1= not useful, 7=extremely useful). There were a total of 48 responses, with 23 of these from first learners (who watched their peer following their own performance) and 25 from second learners (who observed their peer perform the procedure before doing so themselves). The Likert scale ratings for first and second learners are presented in Fig. 5.

**Fig. 6:**
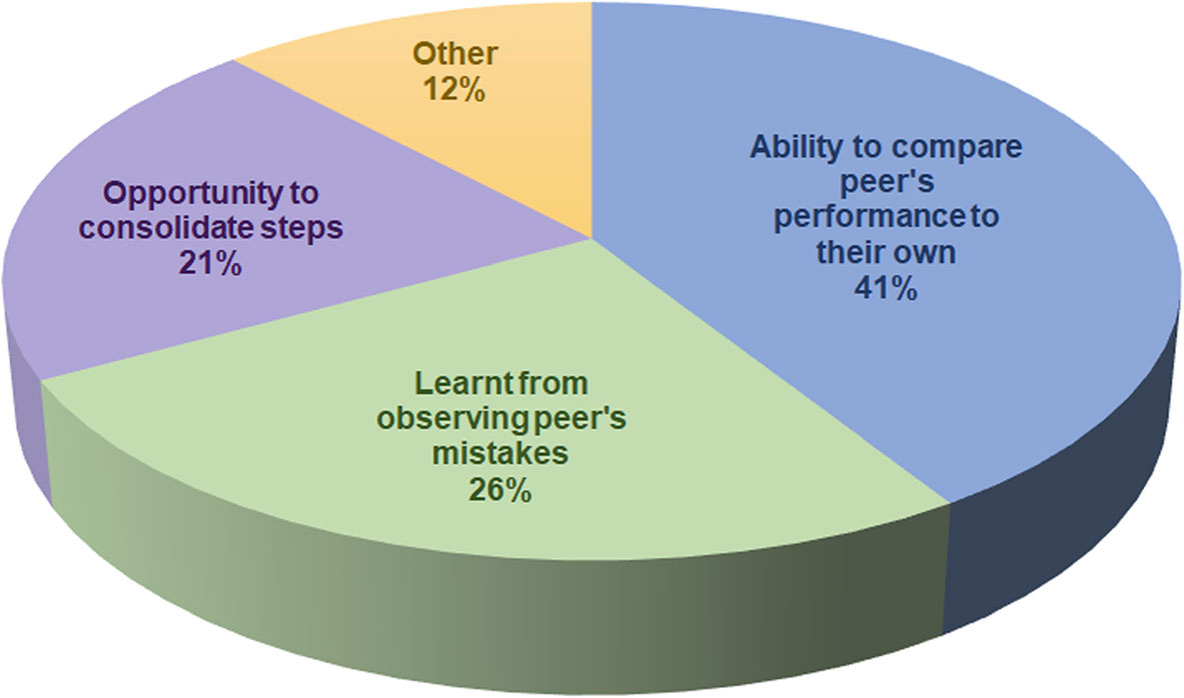
Themes indicating why the peer observation during ‘assessment and feedback’ was considered useful

Thematic analyses of qualitative data from both first and second learners indicated three main reasons for considering peer observation to be useful (Fig. 6). Examples of learner quotes, according to the themes to which they were allocated, are shown in Table 2.

**Table 2:**
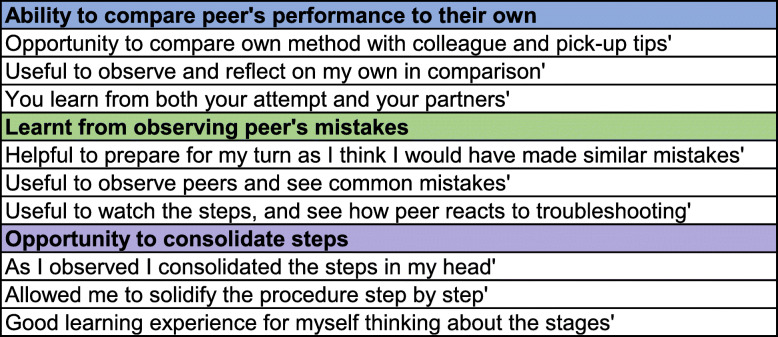
Peer Observation during ‘assessment and feedback’: examples of learner quotes categorised by theme

**Fig. 7:**
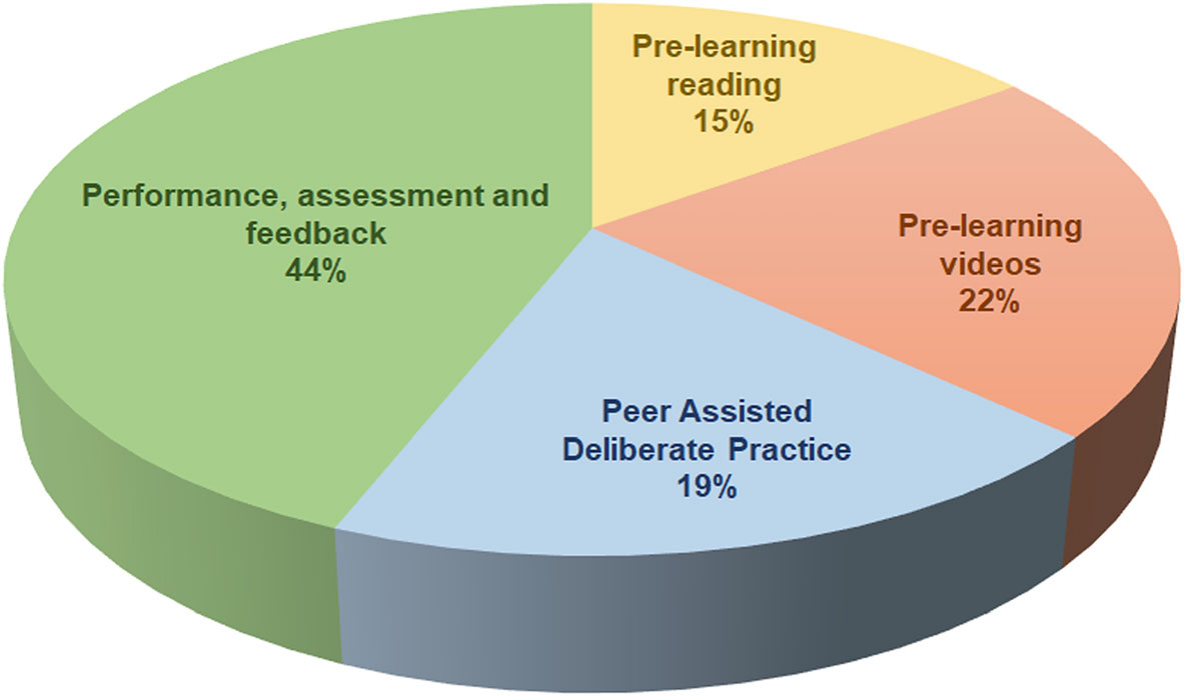
Most valuable section of the SBML programme as selected by learners

The learners were asked which of the four sections of the SBML programme they found to be most valuable and the results are shown in Fig. 7.

## Discussion

This study describes an enhanced approach to SBML and its impact on learners. The traditional SBML approach [1] which has been demonstrated to be effective is, however, limited in its application as it is resource intensive and requires a high ratio of faculty to learners. The enhancements described above were introduced with the intention of optimising the educational impact and generating data to guide the further development of the programme. The SBML approach has been specifically adapted to include additional learning components which permit the scaling up of SBML such that it can be delivered to a large number of postgraduate doctors in the context of a national educational programme.

The pre-learning component was embedded into the SBML approach so that learners were as prepared as possible for the session, and this was considered a valuable part of their experience. The self-paced nature of the online learning experience increases retention rates of the material and learners can repeat parts if they wish [23]. Learners are also able to ‘integrate the knowledge into existing structures in a way that is difficult in a class room’ [23]. Multimedia e-learning has the advantage of easier updating and standardisation. It also allows widespread distribution and easier access for learners. Learners have control over the time and pace of exploring the content and this is a desirable feature for an educational programme which is aimed at postgraduate doctors managing their learning around shift work. The results presented demonstrate that both sections of the pre-learning component were highly rated by learners.

The peer learning component comprised two sections the first of which was peer-assisted deliberate practice. Deliberate practice is a fundamental part of SBML, allowing learners the opportunity to focus on their procedural technique [4]. Barsuk et al. [5] demonstrated that providing learners with time for deliberate practice and feedback during SBML LP sessions produced consistent skill improvement and reduced performance variability within the group.

The theory underpinning this, when applied to SBML sessions for central venous catheter insertion, translated into improved performance in a clinical setting and reduced arterial punctures [2]. To our knowledge, peer-assisted deliberate practice has not been previously employed in the context of a SBML programme. The results shown indicate that learners found the peer-assisted deliberate practice section of the programme described to be useful (Fig. 3). Qualitative data which was subjected to a thematic analysis confirmed that the exchange of experiences and sharing of useful tips and clarification of uncertainties were important aspects of this section. It is possible that these aspects in particular reflect the advantage of working with a peer. However, the familiarisation with equipment and rehearsal of procedure may simply be attributable to deliberate practice itself. Learner quotes relating to stress-free practice do indicate that the programme design supported psychological safety; however, the precise role of peer learning here is uncertain. Examples of learner quotes are provided to substantiate the selected themes and to give an insight into the experiences of learners (Table 1).

The second section of the peer learning component was peer observation of assessment and feedback. Several studies outwith SBML have demonstrated the positive impact of observation in learning motor skills [24–26]. Rohbanfard and Proteau [27] described a mixed model of observation where learners observed both expert and novice performances allowing them to develop a mental template of the process as well as active skills in error detection and a means to correct them. The enhanced SBML programme incorporated this concept in the peer observation section. Huang et al. stated that groups of learners that participated in peer-assisted learning via an online digital platform reported that learning about their peer’s challenging experiences helped them to avoid making the same mistakes themselves [28]. It was initially thought when implementing the enhanced programme that only the second learners would find peer observation useful as the first learners had already performed the procedure and been assessed prior to observing their peer. However, this was not found to be the case as the results indicate that both groups benefitted to a similar extent (Fig. 5). The results of this study align with the work of Rohbanfard and Proteau who reported the benefit of observing novices as well as experts. The qualitative data presented suggest that this is because participants were learning from observing their peer’s mistakes and benefitting from the opportunity to compare their peer’s performance to their own (Fig. 6).

SBML was implemented within an intern boot camp by Cohen et al. [6]. The boot camp involved teaching five clinical skills over a 3-day period and allowed individualised training and assessment of competence prior to interns performing the procedures on patients. Our boot camp builds on this methodology by streamlining the face to face skills lab session whilst enhancing our programme by the addition of pre-learning and peer learning.

Our data suggest that all sections of the enhanced programme are highly rated by the learners. The most valuable section was identified as participation in and observance of assessment and feedback. Interestingly, the other sections of pre-learning reading, pre-learing videos and peer assisted deliberate practice were valued similarly (Fig. 7). The standard mastery performance, assessment and feedback process is key but the additional aspects of our programme also enhanced the learning experience.

It has been demonstrated that the enhanced SBML programme has been positively rated by learners and also generated data which can be used to inform future developments. This enhanced programme can be applied to a large number of learners in a cost-effective manner on a national scale.

The programme is limited by the fact that it lacks an objective measurement of improvement in procedural technique. A further limitation is that the programme has not yet been demonstrated to translate into improved clinical practice. Although real-life clinical performance was not measured, by retaining the core elements of traditional SBML methodologies, the existing translational outcome data generated by McGaghie et al. [1] infer that this approach should impact positively on real life performance. We also acknowledge that the results of this study may be biased by a perceived hierarchy between the learners and researchers and a desire for the learners not to appear ungrateful.

Future studies should examine the basis of the usefulness of the peer learning component of the programme with a view to further developing the innovations introduced. The development of a reliable method for measuring improvement of procedural technique following the pre-learning component would be beneficial.

## Conclusion

SBML is an effective methodology for procedural skill acquisition but has a limited application because of its relatively resource intensive design. The programme described retains the principles of SBML with the addition of pre-learning and peer learning components which are educationally enhancing and allow its application on a national scale. A learning programme for postgraduate doctors has been created which supports training in a psychologically safe environment, is amenable to a range of personal learning needs and was found to be useful by learners.

## Supplementary Information

**Additional file 1.** Enhanced SBML Overview.

**Additional file 2.** Mastery Procedural Pathway.

**Additional file 3.** IMT Boot Camp LP Session Checklist.

**Additional file 4.** Post Course Questionnaire.

**Additional file 5.** Dataset.

## Data Availability

The datasets supporting the conclusions of this article are included within the article and its additional files.

## Abbreviations

SBML: Simulated-based mastery learning
IMT: Internal medicine training
NHS: National Health Service
LP: Lumbar puncture
