# Supplementary figures and images for: An enhanced approach to simulation-based mastery learning: optimising the educational impact of a novel, National Postgraduate Medical Boot Camp
Source: Adv Simul (Lond). 2021 Apr 26;6:15. doi: 10.1186/s41077-021-00157-1 (PMC8074238; doi:10.1186/s41077-021-00157-1)

## Appendix 1: ENHANCED SBML OVERVIEW

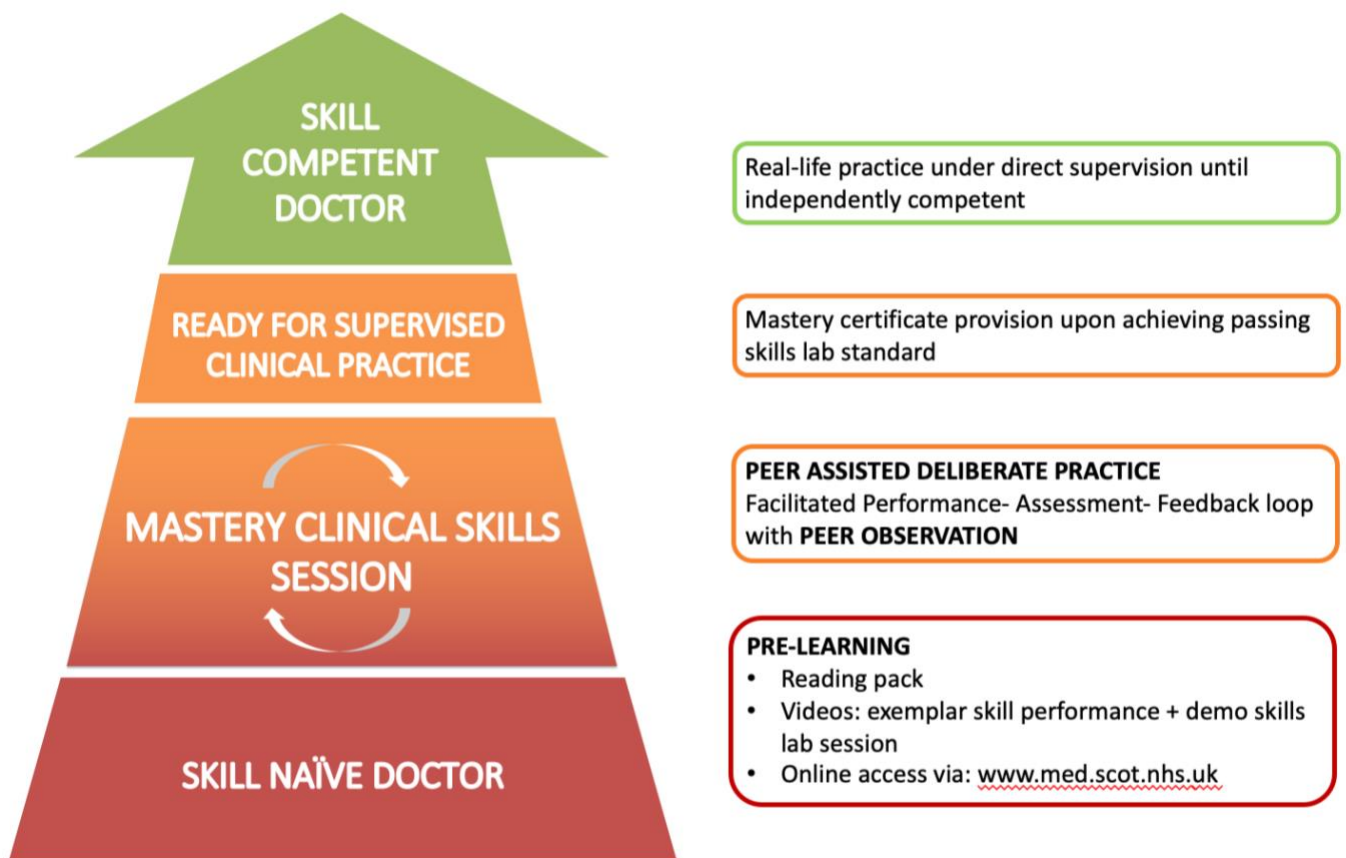

Supplement: Supplementary file 1 — Additional file 1. Enhanced SBML Overview. [file 41077_2021_157_MOESM1_ESM.pdf]

## Appendix 2: MASTERY PROCEDURAL PATHWAY

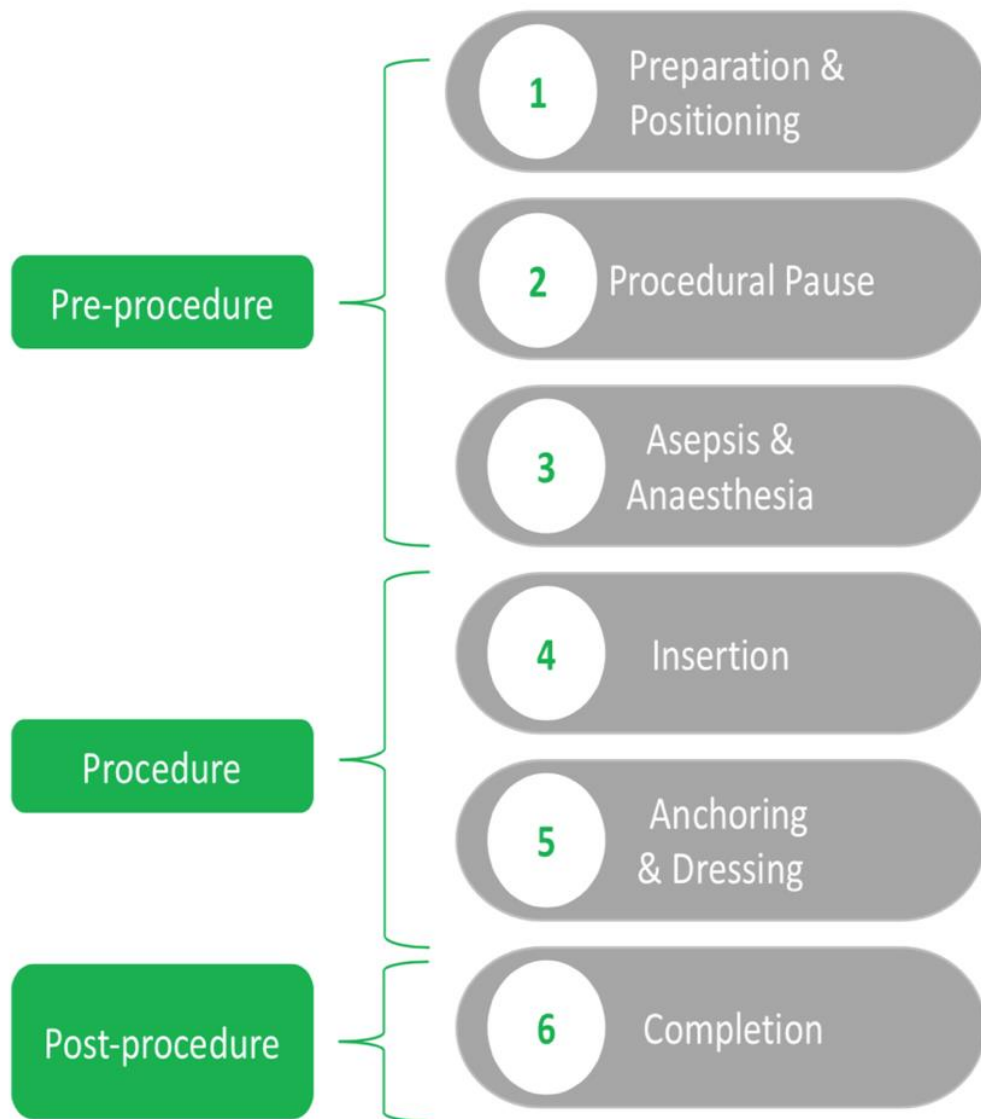

Supplement: Supplementary file 2 — Additional file 2. Mastery Procedural Pathway. [file 41077_2021_157_MOESM2_ESM.pdf]
