# Supplementary material for: An enhanced approach to simulation-based mastery learning: optimising the educational impact of a novel, National Postgraduate Medical Boot Camp
Source: Adv Simul (Lond). 2021 Apr 26;6:15. doi: 10.1186/s41077-021-00157-1 (PMC8074238; doi:10.1186/s41077-021-00157-1)
Supplement: Supplementary file 4 — Additional file 4. Post Course Questionnaire. [file 41077_2021_157_MOESM4_ESM.pdf]

Appendix 4
POST COURSE QUESTIONNAIRE

Learner Experience of Mastery Learning at IMT Bootcamp

Thank you for engaging with the Mastery Pathway. We hope you are enjoying learning your new skills.

This Mastery Pathway is novel and we are keen to understand the value of each component. Please take a few moments to consider the statements below; circle and comment as appropriate.

1. ***How useful was the peer-assisted deliberate practice phase (start of your skill session)?***

Not useful *Extremely useful*
1 2 3 4 5 6 7

Can you please explain why you gave your score?

2. ***Were you the first or second performer within your pairing*?**

First *Second*

3. ***How useful did you find the experience of observing your peer go through the simulated performance-feedback-assessment process?***

Not useful *Extremely useful*
1 2 3 4 5 6 7

Can you please explain why you gave your score?

4. ***What did you think was the most valuable component of the overall Mastery Pathway? (please circle as many as you like)***

Pre-learning reading *Pre-learning videos*

Peer-assisted deliberate practice *Performance-feedback-assessment*

Can you please explain why you chose as you did?

Thank You
